# The influence of attachment style on interpersonal learning in substance use psychotherapy groups

**DOI:** 10.1007/s44202-021-00016-0

**Published:** 2022-01-11

**Authors:** Melina Skentzos, Anissa Naeli, Anastasia Hronis

**Affiliations:** Graduate School of Health, University of Technology Sydney, Ultimo, Australia

## Abstract

Substance Use Disorders (SUDs) are prevalent, long-term conditions, commonly treated via group intervention. Additionally, interpersonal learning (IL) is a therapeutic factor unique to group treatment programs, which has been associated with successful group therapy outcomes. While previous research has suggested attachment styles may predict experiences of IL within groups, research in this area is limited. Therefore, this study aims to investigate if attachment style impacts IL, within SUD group treatment programs and specifically, if anxious and avoidant attachment styles influence IL. Participants (*N* = 38) were recruited voluntarily from an outpatient SUD open group therapy program at a private mental health hospital in Sydney. All participants completed paper-based self-report questionnaires to assess attachment style and IL. Two separate Multiple Linear Regressions (MLRs) revealed anxious attachment was not a significant predictor of IL and avoidant attachment was a significant, negative predictor of IL. Additionally, thematic analysis of qualitative data revealed themes and behaviours which may improve IL. Thus, future directions and implications of the study’s findings indicate the need to conduct additional research into members attachment-based needs to enhance SUD group treatment outcomes.

## Background

Substance Use Disorders (SUDs) are complex conditions, defined as the uncontrolled and continued use of a substance, despite causing clinically significant distress or impairment in daily functioning [1]. SUDs are typically long-term conditions, involving multiple unsuccessful attempts to decrease or discontinue substance use [2]. With one in 20 Australians estimated to experience a substance abuse problem, SUDs can involve and are not limited to alcohol, cannabis, hallucinogens, opioids, stimulants, and tobacco [1, 3]. The use of alcohol and other drugs in Australia has a significant social, economic and health impact at the individual, family and community level. A common treatment approach for SUDs are group therapy programs, with more than 90% of treatment facilities offering group interventions [4, 5]. Thus, cognitive-behavioural therapy (CBT) and coping skills training group interventions have demonstrated empirical support [2, 3].

Despite the prevalence of SUD group interventions, the majority of clinical research has primarily concentrated on individual psychotherapy [4]. However, SUD group interventions have demonstrated equal effectiveness to individual psychotherapy in providing meaningful change for numerous substance addictions [4, 6, 7]. This was consistent with Coco and colleagues [2] who found SUD group treatment was effective in promoting sobriety for numerous substances, when compared to no-treatment control groups and individual psychotherapy. Thus, research suggests the presence of therapeutic factors, unique to group treatment programs which can facilitate change.

### Interpersonal learning

Yalom [7] identified 11 therapeutic factors which inform group treatment and account for therapeutic change, one of those being interpersonal learning (IL) [8]. Yalom and Leszcz [9] define IL as a complex key factor in which group members interact and share aspects of their emotions, thoughts, and perceptions, as well as receive feedback from group members in a safe and collaborative setting [7, 9, 10]. Yalom and Leszcz [9] further conceptualise IL using a dichotomous framework, IL input and IL output. IL input refers to group members learning how others perceive them via group member feedback and IL output refers to behaviour change, in which group members apply feedback provided by others [9]. Thus, Yalom and Leszcz [9] suggest IL affords group members opportunities to learn about relationships and challenge interpersonal distortions, via group member feedback [6, 9, 10].

Numerous studies have demonstrated the importance of IL for group therapy outcomes across different clinical populations [11–13]. IL has been found to lead to improvements in interpersonal skill, including increased awareness of interpersonal behaviours, and increased capacity to give and receive feedback [13, 14]. Additionally, IL has been associated with improved treatment outcomes within Cognitive Behaviour based group therapy programs for panic disorder and social phobia [11, 12]. Moreover, IL output has been associated with significantly lower relapse rates within SUD group therapy, compared to SUD individual therapy [14]. However, findings are mixed with some research suggesting psychotherapy groups can be anxiety provoking and therefore, may hinder interpersonal skill development [15].

### Attachment

Additionally, research suggests that attachment style may predict group members experiences of IL [10, 16]. Belonging is an intrinsic human need which is arguably necessary for survival [9, 16, 17]. Attachment theory is an evolutionary based system which stipulates individuals seek proximity to significant others in times of need and that this behaviour begins with infants seeking proximity to caregivers [16, 18]. Attachment is conceptualised using a dichotomous framework of secure and insecure attachment [16]. Adults with secure attachment are likely to demonstrate satisfactory emotion regulation, secure interpersonal relationships, and a positive view of the self [16]. Contrastingly, adults with insecure attachment typically experience difficulties regulating emotions and may struggle to maintain healthy relationships [16]. Insecure attachment can be further divided into attachment anxiety and attachment avoidance [17]. Anxious attachment is characterised by a need for closeness, chronic concern with partner responsiveness and a fear of rejection [16]. Additionally, avoidant attachment is characterised by discomfort or avoidance of intimacy, suppression of emotion and withdrawal from others [19]. Scoring highly on anxious or avoidant attachment indicates an insecure attachment presentation, whilst lower levels of both indicates more secure attachment.

Furthermore, securely attached group members are likely to perceive relationships with others positively [20]. In contrast, those with high anxious attachment are likely to hold a positive view of other members and a negative internal view of self and those with high avoidant attachment are likely to hold a negative view of others, and a positive view of self [20]. Thus, for insecurely attached individuals maladaptive internal working models may result in a self-fulfilling prophecy, in which individuals anticipate others will respond in a particular manner and resultingly, behave in a manner that produces this behaviour [16]. For example, those high on anxious attachment may inadvertently push others away due to consuming fears of abandonment [20]. Moreover, those with insecure attachment may often misread others intentions and miss opportunities for genuine interactions that would foster security [16, 21]. However, interpersonal feedback may correct interpersonal distortions and provide a context for supporting authentic connection [10, 20]. Thus, the group may act as a social microcosm which provides members with opportunities for corrective emotional experiences [9].

### Attachment and IL

Some studies have presented mixed findings when examining how attachment style impacts IL [10, 22, 23]. Smith and colleagues [23] posit individuals high on either anxious or avoidant attachment demonstrated lower engagement in group activities, greater negative evaluations of the group and lower perceived group support. However, Chen and Mallinckrodt [22] found only those with avoidant attachment were less engaged with the group and less accurate in appraisal of group members interpersonal traits. This was consistent with Shaver and colleagues [24] who suggested avoidant group members are likely to experience difficulty reflecting on their own and others interpersonal behaviours due to the deactivation of their attachment needs. Additionally, Gallagher and colleagues [10] indicated individuals higher in anxious attachment are likely to experience more IL, compared to those lower in attachment anxiety.

### Attachment and SUDs

Moreover, research has demonstrated better overall treatment outcomes for securely attached individuals [17]. However, insecure attachment is more common within SUD groups, compared to non-clinical populations [21, 25]. While insecure attachment is therefore associated with higher rates of substance dependence, this highlights how attachment style may impact group therapy outcomes ([10, 26]. Additionally, attachment influences the severity of substance dependence and predicts the likelihood individuals will seek or complete treatment, with those higher in anxious attachment more likely to seek and complete treatment for SUDs, compared to those with avoidant attachment [25, 27]. Finally, poorer treatment outcomes for anxiously attached individuals, compared to those with avoidant attachment suggest adopting an attachment informed group intervention may be most effective for adults high on attachment anxiety [10].

### The current study

While there exists some theoretical and empirical evidence which highlights the importance of IL in successful group treatment, inconsistencies within the literature, combined with a limited number of studies examining SUD clinical populations, highlights the need to further investigate the role of IL for SUD group psychotherapy.

It is currently unclear what sort of impact attachment style may have on IL and the subsequent outcomes for substance use recovery. Specifically, the research presents mixed findings when examining how anxious attachment style and avoidant attachment style may influence an individual’s experience of IL, within the group therapy context, and which attachment style promotes better IL. The current study aimed to address the identified gaps in the literature, investigating how anxious attachment and avoidant attachment influence IL.

It was hypothesised that (1) attachment style would significantly predict IL in substance-use psychotherapy groups, (2) attachment anxiety would positively predict IL, and (3) attachment avoidance would negatively predict IL.

## Method

### Participants

38 participants took part in the study. All participants were attendees of an outpatient abstinence-based SUD open group therapy program at a private mental health hospital in Sydney. Inclusion criteria required participants to provide informed consent, be aged 18 years or above, be fluent in English and have a SUD diagnosis, as diagnosed by a psychiatrist.

Participants were aged 19 years to 65 years (*M* = 42.16, *SD* = 13.08) and the sample consisted of 20 males (52.63%), 17 females (44.74%) and one participant who preferred not to disclose gender (2.63%). Ethnicity varied with 36.84% (*n* = 14) of the sample identifying as Australian, 2.63% (*n* = 1) New Zealander, 2.63% (*n* = 1) Asian, 2.63% (*n* = 1) Indian, 13.16% (*n* = 5) Middle Eastern, 15.79% (*n* = 6) European, 5.26% (*n* = 2) South American and 21.05% (*n* = 8) mixed ethnicity.

Data collected on the sample’s income bracket suggested a majority earnt above the Australian average, with 2.63% (*n* = 1) earning between $0-$20 000, 2.63% (*n* = 1) earning between $20,000 and $34,999, 15.79% (*n* = 6) earning between $35,000 and $49,999, 28.95% (*n* = 11) earning between $50,000 and $74,999, 23.68% (*n* = 9) earning between $75,000 and $99 999 and 26.32% (*n* = 10) earning over $100,000. Education varied with 2.63% (*n* = 1) leaving school before Year 10, 13.16% (*n* = 5) graduating highschool, 23.68% (*n* = 9) completing TAFE/College, 34.21% (*n* = 13) completing Bachelor’s degrees, 13.16% (*n* = 5) completing Master’s degrees and 2.63% (*n* = 1) completing a Doctorate.

### Materials

#### Participant demographics

Information was gathered about participant demographic variables, including gender, age, ethnicity, relationship status, education, and income bracket. Information on therapy specific variables including substances of addiction, previous number of sessions attended, and group size was also gathered. Gender required a single response using multiple choice options (e.g., male, female, non-binary) as did ethnicity (e.g., Australian, Indigenous Australian, Asian) and relationship status (e.g., single, in a relationship, de-facto). Age, substances of addiction and number of sessions attended required an open text response.

#### Kessler-6 (K-6, [28])

A 6-item self-report measure assessing individual’s psychological distress over the past 30 days (e.g. “about how often did you feel…nervous?…hopeless?…worthless?). The K-6 uses a 5-point Likert scale which ranges from 5 (All of the time) to 1 (None of the time). Response scores are summed to achieve a total score, with scores between 6–18 indicating no probable serious mental illness and scores between 19–30 indicating probable serious mental illness [28]. The measure is reported to have good test–retest reliability and internal consistency [29].

#### Qualitative questions on interpersonal learning

Participants were provided with a description of interpersonal learning and were asked to respond to two qualitative questions, “What do you think leads to better interpersonal learning?” and “Why is it important to you to experience interpersonal learning?”.

#### Attachment Style Questionnaire- Short Form ([ASQ-SF, [30])

A 29-item self-report measure assessing attachment style on two domains, attachment anxiety (e.g. “It’s important to me that others like me”) and attachment avoidance (e.g. “I prefer to depend on myself rather than other people”). Each item was rated on a 6-point Likert scale ranging from 1 (Totally Disagree) to 6 (Totally Agree). Seven items were reverse scored so that a higher score was indicative of more insecure attachment across both subscales. Total scores were calculated by averaging the responses assigned to each subscale. The ASQ-SF has good reliability and validity [31]. The ASQ-SF is based on the Attachment Style Questionnaire (ASQ, [32]) which has also been found to have good reliability and validity [33].

#### Therapeutic factors inventory- short Form ([TFI-S; [34])

A 23-item self-report measure which assesses the presence of Yalom’s [7] therapeutic factors on four domains. These include instillation of hope (e.g. “things seem more hopeful since joining group”), secure emotional expression (e.g. “I feel a sense of belonging in this group”), awareness of relational impact (e.g. “in group I’ve really seen the social impact my family has had on my life”) and social learning (e.g. “in group sometimes I learn by watching and later imitating what happens”). Each item is rated on a 7-point Likert scale which ranges from 1 (Strongly Disagree) to 7 (Strongly Agree). Due to the awareness of relational impact factor assessing interpersonal learning input and the social learning factor assessing interpersonal learning output, total scores for the two subscales were calculated [35]. Total scores were obtained by summing all item ratings and multiplying by a factor score weight, with higher scores indicating more interpersonal learning. The TFI-S is based on the Therapeutic Factors Inventory (TFI, [8]) which was found to have good reliability and validity [34]. The TFI-S has also demonstrated good internal consistency and good convergent and predictive validity [35].

### Procedure

This study was approved by the Human Research Ethics Committee (ETH21-5765; #1763). Participants were recruited voluntarily from the abstinence-based group therapy program. The therapy groups were run by psychologists independent to the current research project. The approach to treatment was Cognitive Behaviour Therapy.

### Data analysis

Two Multiple Linear Regressions (MLR) to determine if attachment style significantly predicted IL within SUD psychotherapy groups. For the first MLR the independent variable (IV) was ‘attachment style’ which consisted of two levels: attachment anxiety and attachment avoidance. The dependent variable (DV) was social learning. The second MLR retained ‘attachment style’ as the IV and employed awareness of relational impact as the DV. All quantitative analyses were conducted with a conventional alpha level set at 0.05 a priori [36]. Thematic analyses were conducted on qualitative responses by the author. Responses were coded according to themes, and these were cross checked by a colleague.

## Results

### Descriptive statistics

Participants reported on their substance(s) of addiction. See Table 1. Moreover, 68.42% (*n* = 26) reported addiction to one substance, 18.42% (*n* = 7) reported addiction to two substances, 10.53% (*n* = 4) reported addiction to three substances and 2.63% (n = 1) reported addiction to four substances.

**Table 1:** Participants substances of addiction

Substances	%	*n*
Alcohol	97.37	37
Marijuana	18.42	7
Cocaine	10.53	4
Gambling	7.89	3
Benzodiazepine	2.63	1
Opioid	2.63	1
Methamphetamine	2.63	1
Prescription Medication	2.63	1
Pain Medication	2.63	1

%, percentage of sample; n, number of participants from sample

Participants reported on psychological distress as measured by the K6. Participants’ scores ranged from 8 to 28 (*M* = 18.26, *SD* = 5.11). Data was also collected about group size for the day of study participation and the number of group therapy sessions a participant had previously attended. Group size varied, ranging from 6 to 14 participants (*M* = 9.18, *SD* = 2.31). The number of previously attended group sessions also varied across participants with 47.37% (*n* = 18) attending between 1 and 10 sessions, 26.32% (*n* = 10) attending 11–20 sessions, 7.89% (*n* = 3) attending 21–30 sessions, 2.63% (*n* = 1) attending 31–40 sessions, 13.16% (*n* = 5) attending 41 + sessions and 2.63% (*n* = 1) not providing a response. This data was collected with the intention of running a moderation analysis to explore whether client and group characteristics influence the relationship between attachment and IL. However, due to sample size this was unable to be completed and therefore, only descriptive statistics are reported.

Additionally, participant scores on attachment anxiety ranged from 1.15 to 5.85 (*M* = 3.43, *SD* = 1.00). Participant scores on attachment avoidance ranged from 1.44 to 5.06 (*M* = 3.32, *SD* = 1.01). For interpersonal learning, participant scores on social learning ranged from 2.91 to 5.09 (*M* = 4.13, *SD* = 0.63). Participants scores on awareness of relational impact ranged from 2.70 to 4.79 (*M* = 3.91, *SD* = 0.59).

### Data diagnostics

Data was screened for data entry errors and missing data via visual inspection. Due to one participant failing to provide a response to item 19 of the ASQ-SF, this single missing item was imputed by averaging the participants’ responses on the attachment anxiety subscale. All 38 participants were retained in the final sample and a power analysis was conducted to determine whether the final sample was sufficient to detect a large effect size. The analysis indicated that a sample size of 38 participants was sufficient to detect an effect size of 0.8 in a linear regression model, with a beta coefficient of 0.42 and an *R*^2^ of 0.17.

Prior to running the main analyses, the data was screened to ensure assumptions were met. The assumption of independence was met due to independence of observations being implicit within the studies design. Linearity and the presence of outliers was assessed through partial regression plots and plots of the studentized residuals against the predicted values. Visual inspection of plots indicated all relationships were linear and no outliers were present, as no cases were greater than three standard deviations from the mean, therefore both assumptions were met. Homoscedasticity was assessed through visual inspection of plots of studentized residuals versus unstandardised predicted values. As residuals appeared randomly spread, the assumption was met. There was no evidence of multicollinearity, as assessed by tolerance values greater than 0.1, therefore the assumption was met. Normality was assessed through visual inspection of histograms of residuals which indicated the data was normally distributed, therefore the assumption was met.

### Main analysis

#### MLR social learning

The first MLR was run to predict social learning from attachment anxiety and attachment avoidance. The multiple regression model statistically significantly predicted social learning F(2, 35) = 5.40, p = 0.009. Attachment anxiety was not a significant predictor of social learning (*p* = 0.615), while attachment avoidance was a significant negative predictor of social learning (*p* = 0.005). Regression coefficients and standard errors can be found in Table 2.

**Table 2:** Multiple regression results for Social Learning

Social learning	B	95% CI for B		*SE B*	*p*	*R*^*2*^	*R*^*2*^_adjusted_
		*LL*	*UL*				
Model					0.009**	0.24**	0.19**
Constant	5.04***	4.29	5.78	0.37	< 0.001***		
Attachment anxiety	0.06	− 0.17	0.29	0.11	0.615		
Attachment avoidance	− 0.33**	− 0.56	− 0.11	0.11	0.005**		

Model = “Enter” method in SPSS statistics; *B*,  unstandardized regression coefficient; CI, confidence interval; *LL*, lower limit, *UL*, upper limit, *SE B*, standard error of the coefficient; p, p-value statistical significance; *R*^*2*^ = coefficient of determination; *R*^*2*^_adjusted_, adjusted *R*^*2*^

*p < 0.05. **p < 0.01. ***p < 0.001

#### MLR awareness of relational impact

The second MLR was run to predict awareness of relational impact from attachment avoidance and attachment anxiety. The multiple regression model statistically significantly predicted awareness of relational impact F(2, 35) = 5.72, p = 0.007. Attachment anxiety was not a significant predictor of awareness of relational impact (*p* = 0.796), while attachment avoidance was a significant negative predictor of awareness of relational impact (*p* = 0.006). Regression coefficients and standard errors can be found in Table 3.

**Table 3:** Multiple regression results for Awareness of Relational Impact

Awareness of Relational Impact	*B*	95% CI for *B*		*SE B*	*p*	*R*^*2*^	*R*^*2*^_adjusted_
		*LL*	*UL*				
Model					0.007**	0.25**	0.20**
Constant	4.82***	4.13	5.51	0.34	< 0.001***		
Attachment anxiety	0.03	− 0.19	0.24	0.10	0.796		
Attachment avoidance	− 0.30**	− 0.51	− 0.09	0.10	0.006**		

Model = “Enter” method in SPSS statistics; *B*, unstandardized regression coefficient, CI, confidence interval; *LL*, lower limit; *UL*, upper limit; *SE B*, standard error of the coefficient; p, p-value statistical significance; *R*^*2*^,  coefficient of determination; *R*^*2*^_adjusted_, adjusted *R*^*2*^

*p < 0.05. **p < 0.01. ***p < 0.001

### Qualitative analysis

Of the sample, 68.42% (n = 26) provided qualitative responses to the question “What do you think leads to better interpersonal learning?” and 57.89% (n = 22) of participants provided qualitative responses to the question “Why is it important to you to experience interpersonal learning?”. Thematic analysis was conducted on IL responses and the following five themes were identified (a) sharing skills and learnings, (b) sharing emotions, (c) feedback, (d) implementing learnings, and (e) inclusivity, support, and respect.

### Sharing skills and learnings

Some participants reported sharing practical strategies and resources to manage addiction was important for developing IL within the group. Specifically, participants reported sharing their own personal experiences and knowledge of addiction with the group would lead to better IL, as participants are learning skills from other participants. For example one participants stated, “people share what works for them”. Participants reported by sharing resources, other participants capacities to manage addiction will improve. For example, another participant stated, “share strategies, books, podcasts”. Many participants reported IL was important as it allows for “better recovery outcomes”, less frequent relapses and removes the isolation of addiction by offering multiple perspectives on how to manage it.

### Sharing emotions

Participants reported sharing and listening to others talk about their feelings leads to better IL. For example “tell people how they make you feel” and “talk about your feelings”. Participants reported it was important to share emotions and be vulnerable with the group, as this provides opportunities for learning from others, and provides a safe space for participants to express themselves. Participants noted more learning could occur if all members participate and share with the group, due to this fostering an open and supportive group environment. For example “if we do this then we all feel supported and like we can share openly in group”.

### Feedback

Participants reported it was important to give, receive and reflect on feedback provided by group members and facilitators, as this leads to better IL. For example, “get feedback on yourself and how you treat other people”. Additionally, participants reported engaging in active listening while others are providing feedback may result in better IL. Participants reported reflecting on feedback would allow group members to “discern what is/isn’t applicable to the individual”, and therefore, how feedback could be applied. Participants reported this would encourage learning on multiple levels including learning about the self, learning about addiction and learning how others perceive you and how you impact others. For example, “we can change our behaviour and learn how we impact other people in group”.

### Implementing learnings

Participants reported it was important to implement learnings from the group as this leads to behaviour change. For example, “try what the facilitators tell us”. Participants reported taking on feedback from the group was important for the purposes of behaviour change and improving IL. For example, “willingness to try what is suggested”. Participants reported applying strategies discussed in group and sharing the outcome of their endeavour with the group, leads to better IL.

### Inclusivity, support and respect

Participants reported that treating other group members with kindness and respect leads to better IL, as identified by specific participants quotes such as “be kind and respect each other”. Participants reported this allowed everyone the opportunity to share willingly, without judgement, and to “let everyone speak if they want to”. Participants reported it was important to support others but not necessary to provide direct advice. For example, one participant specified “give each other support and help but not advice”.

## Discussion

Despite prior research highlighting the importance of IL for positive group therapy outcomes, research examining IL and attachment style within SUD group therapy remains limited. The present study examined if attachment style significantly predicts IL within SUD psychotherapy groups. Furthermore, the study aimed to contribute to a deeper understanding of how SUD group-based interventions may improve treatment outcomes, by considering and accounting for members’ attachment needs. Thus, this research may assist to guide future research efforts, as well as inform group therapy practice by providing several practice implications concerning how to accommodate for attachment-based needs. To address research questions two MLRs and thematic analyses were conducted.

### Attachment and IL

Hypothesis one predicted attachment style would significantly predict IL in SUD psychotherapy groups. Results from the first MLR found attachment style significantly predicted social learning, namely IL output [9]. Results from the second MLR found attachment style significantly predicted awareness of relational impact, namely IL input [9]. Therefore, hypothesis one was supported. This supported previous research which suggested internal working models formed during early attachment relationships with caregivers may determine group members interpersonal behaviours within group settings [16]. Therefore, maladaptive internal working models for insecurely attached group members may interfere with IL, due to increasing the likelihood insecurely attached individuals will misread others intentions and miss IL opportunities [16, 20].

### Anxious attachment

Hypothesis two predicted attachment anxiety would positively predict IL, therefore those higher in anxious attachment would report significantly more IL. The first MLR found attachment anxiety did not significantly predict social learning and the second MLR found attachment anxiety did not significantly predict awareness of relational impact. Therefore, hypothesis two was not supported. While the literature was mixed on the direction of the relationship between anxious attachment and IL, prior research indicated anxious attachment would significantly predict IL [10, 16]. Therefore, findings did not support previous research. However, Tasca and colleagues [19] found cohesion within the group is particularly important for anxiously attached group members due to a specific need for closeness and fear of rejection. This is consistent with Yalom and Leszcz [9] who indicated group cohesion is a foundational therapeutic factor which may mediate other factors, including IL. Thus, while examining group cohesion was outside the scope of this study, the literature suggests group cohesion may have confounded results, specifically for anxious attachment. Thus, future research should investigate if anxious attachment predicts IL, whilst controlling for group cohesion. Furthermore, due to limited sample size, the present study did not examine if group characteristics, such as as number of previously attended sessions may impact the relationship between attachment and IL. Due to significant variability within number of previously attended sessions, some participants may have had insufficient time to develop good group cohesion and this may have impacted capacity for IL for those with high anxious attachment [9, 20].

### Avoidant attachment

Hypothesis three predicted attachment avoidance would negatively predict IL, therefore those higher in avoidant attachment would report significantly less IL. The first MLR found attachment avoidance was a significant negative predictor of social learning and the second MLR found attachment avoidance was a significant negative predictor of relational impact. Therefore, hypothesis three was supported. This finding was consistent with prior research which indicated those high on avoidant attachment would engage in less IL due to negative internal working models of others, resulting in reduced capacity to connect and be vulnerable with the group [20, 37]. Therefore, close relationships with other members may be perceived as unnecessary and threatening to those high on avoidant attachment [37]. Furthermore, positive internal working models of self may lead to disinterest in changing interpersonal behaviours [19]. Consequently, these group members are less likely to see positive treatment outcomes from group psychotherapy. This presents important implications for group facilitators as the need to account for member’s attachment needs, in order to encourage IL and subsequently enhance treatment outcomes, is highlighted.

### Qualitative data

Thematic analysis of qualitative data explored participants beliefs about behaviours that lead to better IL**.** The analysis revealed the following five themes: (a) sharing skills and learnings, (b) sharing emotions, (c) feedback, (d) implementing learnings, and (e) inclusivity, support, and respect. Specifically, participants identified the following behaviours within themes: (1) sharing emotions and thoughts with the group, (2) implementing strategies and feedback provided by group, (3) sharing strategies and experiences to manage addiction, (4) allowing every member the opportunity to share, (5) listening to others and (6) reflecting on feedback received. Results were consistent with the literature which indicated receiving feedback and encouraging reflection were associated with better IL [9]. Additionally, qualitative results supported the studies quantitative outcomes as participants reported genuineness and showing vulnerability were important for IL, and results demonstrated avoidant attachment was a significant negative predictor of IL. Thus, results provide further helpful implications for group facilitators through highlighting specific behaviours which lead to improved IL and thereby, improve treatment outcomes [11, 12, 14].

### Limitations and future research

While the present study initially aimed for a sample size of 100 participants, this was unobtainable due to Covid-19 lockdown restrictions limiting participant attendance. Therefore, the study was unable to run additional moderation analyses to explore if client and group characteristics, such as gender or number of previously attended sessions impact the relationship between attachment and IL. Thus, moderation analyses are a direction for future research. Furthermore, the study was limited by its reliance on self-report measures due to the potential for social desirability bias and response bias and failing to control for these. The study also failed to control for extraneous variables such as group cohesion which may have confounded results when investigating if anxious attachment was a predictor of IL. Therefore, future research should re-examine anxious attachment and IL, whilst controlling for group cohesion. Additionally, the studies sample income demographics being higher than the Australian average, and sampling occurring at a private hospital suggests the studies external validity may be limited and not entirely reflective of the general population. Furthermore, external validity may be limited due to all data obtained from an open group program, therefore results may not generalise to closed groups. Finally, the study did not include a specific measure of secure attachment, thus future research should investigate the effects of secure and insecure attachment on IL.

### Implications

Despite these limitations, the present study has added to a growing body of research extending attachment theory to group therapy processes. This study shed light on the role of attachment as a predictor of IL and how this impacts group therapy outcomes. Considering member’s attachment-based needs is important to developing a deeper understanding of members’ interactions within groups. Specifically, the study highlights the need for facilitators to account for high avoidant attachment. However, identification of concrete behaviours that improve IL provides a first step in the direction of deriving practice guidelines for improvement of IL for group facilitators. Future research in attachment and IL are warranted, in order to inform the improvement of treatment outcomes for SUD group interventions.

## Data Availability

The data and code utilised in this study is available on request from the corresponding author. The data is not publicly available due to containing information that could compromise the privacy of research participants.
